# Maternal immunity enhances systemic recall immune responses upon oral immunization of piglets with F4 fimbriae

**DOI:** 10.1186/s13567-015-0210-3

**Published:** 2015-06-23

**Authors:** Ut V Nguyen, Vesna Melkebeek, Bert Devriendt, Tiphanie Goetstouwers, Mario Van Poucke, Luc Peelman, Bruno M Goddeeris, Eric Cox

**Affiliations:** Laboratory of Immunology, Faculty of Veterinary medicine, Ghent University, Salisburylaan 133, 9820 Merelbeke, Belgium; Laboratory of Animal Genetics, Faculty of Veterinary Medicine, Ghent University, Heidestraat 19, 9820 Merelbeke, Belgium; Department of Biosystems, Faculty of Bioscience Engineering, KU Leuven, Kasteelpark Arenberg 30, B-3001 Leuven, Belgium

## Abstract

F4 enterotoxigenic *Escherichia coli* (ETEC) cause diarrhoea and mortality in piglets leading to severe economic losses. Oral immunization of piglets with F4 fimbriae induces a protective intestinal immune response evidenced by an F4-specific serum and intestinal IgA response. However, successful oral immunization of pigs with F4 fimbriae in the presence of maternal immunity has not been demonstrated yet. In the present study we aimed to evaluate the effect of maternal immunity on the induction of a systemic immune response upon oral immunization of piglets. Whereas F4-specific IgG and IgA could be induced by oral immunization of pigs without maternal antibodies and by intramuscular immunization of pigs with maternal antibodies, no such response was seen in the orally immunized animals with maternal antibodies. Since maternal antibodies can mask an antibody response, we also looked by ELIspot assays for circulating F4-specific antibody secreting cells (ASCs). Enumerating the F4-specific ASCs within the circulating peripheral blood mononuclear cells, and the number of F4-specific IgA ASCs within the circulating IgA^+^ B-cells revealed an F4-specific immune response in the orally immunized animals with maternal antibodies. Interestingly, results suggest a more robust IgA booster response by oral immunization of pigs with than without maternal antibodies. These results demonstrate that oral immunization of piglets with F4-specific maternal antibodies is feasible and that these maternal antibodies seem to enhance the secondary systemic immune response. Furthermore, our ELIspot assay on enriched IgA^+^ B-cells could be used as a screening procedure to optimize mucosal immunization protocols in pigs with maternal immunity.

## Introduction

F4 fimbriated enterotoxigenic *E. coli* (F4^+^ ETEC) are one of the main pathogens causing neonatal and post-weaning diarrhoea leading to severe economic losses in the pig industry due to mortality, growth retardation and medication costs. ETEC adhere with their F4 fimbriae to intestinal F4-specific receptors resulting in colonization of the small intestine and release of enterotoxins. Neonatal diarrhoea is prevented by vaccination of sows, which will then protect their offspring by ETEC-specific lactogenic antibodies [1,2]. As at weaning piglets are suddenly deprived of these passive antibodies, active mucosal immunity should be elicited. To induce intestinal immunity, oral immunization is most suited; for instance, oral immunization of F4R^+^ F4-seronegative piglets resulted in the induction of a protective immunity [3]. However, the presence of ETEC-specific neutralizing lactogenic antibodies may interfere with the induction of immune responses to orally administered vaccines [4,5]. Even deprived of milk antibodies in the gut at weaning, maternal IgG is often still present in serum [6]. Some studies showed that maternally derived serum antibodies can suppress the induction of an immune responses [4,7], whereas others claim the potential of such antibodies to prime immunity via bidirectional transport by neonatal Fc receptors (FcRn) on porcine enterocytes [8,9]. Consequently, it remains to be demonstrated if conventionally reared pigs with maternal F4-specific serum antibodies can be orally immunized with F4 fimbriae. The presence of maternal antibodies might interfere with the oral induction of an immune response, and could also hamper the detection of vaccine-induced antibodies via ELISA. Therefore other ways to measure an immune response were explored in this study, using a similar strategy described in Saletti et al. [10]. Results indicate that the combination of an ELIspot assay with immunomagnetic enrichment of IgA^+^ B cells was most sensitive to monitor the immune response upon oral immunization of piglets with soluble F4 fimbriae in the presence of maternal F4-specific serum antibodies and demonstrate an immune response in the animals orally immunized in the presence of maternal antibodies.

## Materials and methods

### Selection of pigs

Fifteen, 3- to 4-week-old, Belgian Landrace x Pietrain piglets were selected from three farms. On two of these farms primiparous and multiparous sows were vaccinated against neonatal ETEC infections using Porcilis Porcoli Diluvac Forte (F4ab, F4ac, F5 and F6). Piglets were screened for the presence of F4-specific serum antibodies and positive animals were tested for the absence of F4-specific antibody-secreting cells (ASCs) to assure that the F4-specific serum antibodies were of maternal origin. Furthermore, all piglets were genotyped for *MUC4* as previously described [11] to evaluate the F4 receptor status. The *MUC4* homozygous and heterozygous genotypes are positive in the in vitro villous adhesion assay for F4ac *E. coli* binding indicating they express the F4acR [11,12].

Four seronegative and 11 seropositive animals, all heterozygous for *MUC4* and without F4-specific ASCs were still suckling when tested. They were weaned and immediately transported to isolation units with water and feed *ad libitum*. To prevent *E. coli* infections upon weaning, animals were treated orally with colistin for five consecutive days (150 000 U/kg body weight/day; ProMycine® Pulvis, VMD, Arendonk, Belgium) until two days before the immunization. Experimental and animal management procedures were approved by the animal care and ethics committee of the Faculty of Veterinary Medicine (EC2010/042).

### Immunization and sampling

The animals were divided into 4 groups, which were housed separately: two groups were orally immunized with 1 mg F4ac fimbriae in 10 mL phosphate buffered saline (PBS) on three consecutive days and once again 14 days post primary immunization (dppi), a group of four piglets without F4-specific maternal antibodies (seronegative-oral group), and a group of six piglets with F4-specific maternal antibodies (maternal-oral group). A third group of two pigs with F4-specific maternal antibodies was intramuscularly immunized with 100 μg F4ac fimbriae emulsified with incomplete Freund’s adjuvant on 0 and 14 dppi (maternal-IM group) and a fourth group of three pigs with F4-specific maternal antibodies received PBS orally (the maternal-PBS group).

The F4ac fimbriae, purified from the enterotoxigenic *E. coli* strains GIS26 (O149:K91, F4ac^+^, LT^+^STa^+^STb^+^), were used for the immunizations, whereas those from strain IMM01 were used to measure the antibody responses in ELISA and ELIspot assays. Isolation and purification of the fimbriae occurred as previously described [13]. Blood was sampled from the external jugular vein at 0, 9, 14, and 21 dppi in tubes with heparin (50 units/mL blood, LEO Pharma N.V/S.A, Belgium) to determine the kinetics of the F4-specific ASCs and without heparin to determine the F4-specific serum antibody response. At 21 dppi animals were euthanized using pentobarbital (Kela, Hoogstraten, Belgium) and villi were collected from a mid-jejunal segment (15 cm) to confirm the F4 receptor status using the in vitro villous adhesion assay as previously described [14].

### F4-specific serum antibody ELISA

Serum samples were heat-inactivated,kaolin-treated and tested in ELISA for F4ac-specific serum antibodies as previously described [14] with slight modifications. A 96-well microtiter plate (Maxisorp, Life Technologies, Merelbeke, Belgium) was coated with 1 μg/mL (in PBS) F4ac-specific monoclonal antibody (mAb; IMM01) [15] or with 5 μg/mL F4ac fimbriae (strain IMM01) to detect total F4-specific antibodies or F4-specific IgG and IgA antibodies, respectively. After 2 h incubation at 37 °C, remaining binding sites were blocked for 1 h at 37 °C with PBS + 0.2% Tween® 80. To detect total F4-specific antibodies, consecutively F4ac fimbriae (GIS26) (25 μg/mL in PBS + 0.2% Tween®20 + 3% bovine serum albumin),two-fold serial dilutions of the sera and cross-adsorbed HRP-conjugated anti-pig IgG antibody (Bethyl Laboratories, Montgomery, TX, USA) were each added for 1 h at 37 °C. To detect F4-specific antibody subclasses, consecutively the treated sera, mouse anti-swine IgG or IgA mAbs (clones 27.8.1 and 28.4.1, respectively; [16]) and HRP-conjugated anti-mouse IgG antibody (Bethyl Laboratories) were each added for 1 h at 37 °C. Between each incubation step plates were washed three times with PBS + 0.2% Tween®20 (PBS-T). Then, ABTS (Roche, Mannheim, Germany) was added and after 30 min at 37 °C the optical density was measured at 405 nm. The cut-off values were calculated as the mean optical density at 405 nm of sera from all F4-seronegative pigs at day 0 increased with 3 times the standard deviation. The titer was the inverse of the highest dilution with an optical density higher than the calculated cut-off value.

### Enrichment of circulating IgA^+^ B-cells

Peripheral blood mononuclear cells (PBMC) were isolated from 20 mL heparinized blood by density gradient centrifugation on Lymphoprep® (Axis-Shield, Oslo, Norway) [14]. The interphase was collected and remaining erythrocytes were lysed with ammonium chloride (0.8%, w/v) for 10 min. The cells were pelleted (350 g, 10 min, 4 °C),resuspended in PBS with 1 mM EDTA (Sigma-Aldrich, Steinheim, Germany; PBS-EDTA) and the viable cell concentration was determined. To select IgA^+^ cells, 3.0 × 10^8^ PBMCs were incubated on ice for 20 min with biotinylated polyclonal goat anti-pig IgA antibodies (Abcam, Cambridge, UK). Thereafter, cells were washed twice with PBS-EDTA, incubated with mouse anti-biotin mAb-conjugated microbeads (isotype IgG_1_; Miltenyi Biotec, Bergisch Gladbach, Germany) for 15 min on ice and resuspended in 5 mL PBS-EDTA supplemented with 1% fetal calf serum (FCS, Life Technology) (MACS-buffer). The cell suspension was brought into a LS column (Miltenyi Biotec) and placed into a QuadroMACS™ separation unit (Miltenyi Biotec). The IgA^+^ cells were eluted with 5 mL MACS-buffer following removal of the column from the magnetic field. The obtained IgA^+^ fraction, approximately 2-4% of the PBMC, was resuspended at 5.0 × 10^6^ cells/mL in complete medium (RPMI 1640 supplemented with 100 μg/mL kanamycine, 5% FCS, 100 U/mL penicilin and 100 μg/mL streptomycin, 1% non-essential amino acids, 1 mM sodium pyruvate (all from Life Technology), 2 mM L-glutamine (Sigma-Aldrich)).

### Detection of F4-specific ASCs

The number of F4-specific ASCs was determined as previously described [14] with slight modifications. A 96-well microtiter plate was coated with F4-specific mAbs for total F4-specific ASC detection or with F4ac fimbriae for F4-specific IgA^+^ ASC detection as for the ELISA assay. Subsequently, 5.0 × 10^5^ PBMCs or IgA^+^ cells in 100 μL complete medium were added overnight at 37 °C in a humidified 5% CO_2_ atmosphere. Thereafter, the cells were removed by washing 10 times with PBS-T. To detect total F4-specific ASCs, the plates were incubated for 1 h at 37 °C with cross-adsorbed HRP-conjugated mouse anti-pig IgG (1:5000; Bethyl Laboratories). To detect F4-specific IgA^+^ ASCs, the plates were incubated for 1 h at 37 °C with anti-swine IgA mAb (clone 27.8.1) followed by an incubation for 1 h at 37 °C with HRP-conjugated anti-mouse IgG antibody (1:1000; Bethyl Laboratories). To visualize spots, 50 μL 3,3’,5,5’-tetramethylbenzidine liquid substrate (Sigma-Aldrich) was applied for 15 min at room temperature. The resulting spots were scanned with an ImmunoSpot® analyzer (CTL-Europe GmbH, Germany). For each condition spots in 8 wells (5.0 × 10^5^ cells/well) were counted to obtain the number of F4-specific total or IgA^+^ ASCs per 4.0 × 10^6^ PBMCs or F4-specific IgA^+^ ASCs per 4.0 × 10^6^ IgA^+^ cells. Results were normalized to 3.0 × 10^8^ PBMCs, the average number of PBMCs isolated from 20 mL blood.

### Statistical analysis

The differences in F4-specific antibody log2 titers and in numbers of F4-specific ASCs, after square root transformation to homogenize variances, were analyzed between groups at one time point or within a group between different time points. Analysis occurred with GraphPad Prism version 5.0c for Mac OS X using one-way ANOVA with Tukey’s post hoc test. The equal variances of the data were verified by Levene’s test. A *p*-value <0.05 was considered statistically significant. Data are presented as mean ± SEM.

## Results

### Maternal immunity obscures or suppresses serum antibody responses following oral immunization

**Figure 1:**
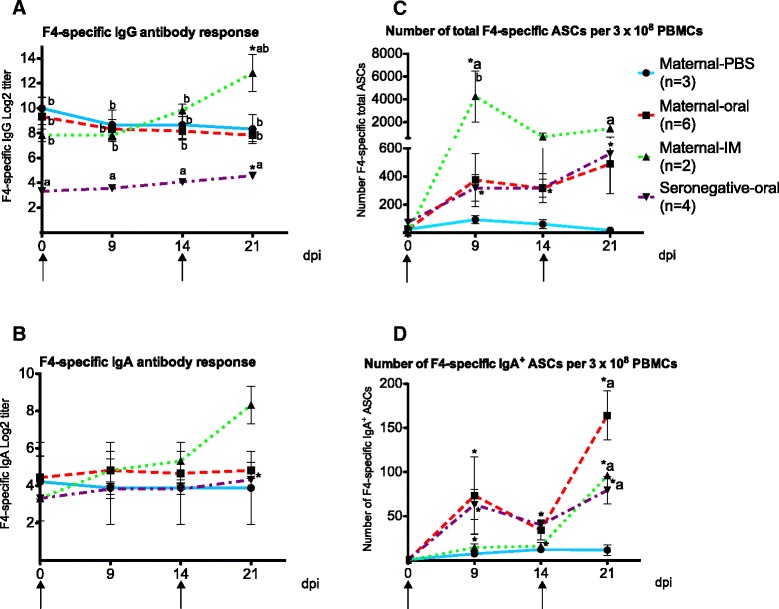
**The immune response of pigs after immunization with F4 fimbriae.** The F4-specific IgG antibody responses (**A**), the F4-specific IgA antibody responses (**B**), number of F4-specifc total antibody secreting cells (ASCs) per 3.0 × 10^8^ peripheral blood monomorphonuclear cells (PBMCs) (**C**), number of F4-specific IgA^+^ ASCs per 3.0 × 10^8^ PBMCs (**D**). Piglets were immunized with F4 fimbriae at day 0 and at 14 days post primary immunization (dppi). Data are presented as the mean ± SEM. The asterisk indicates a statistical significant difference (*p* < 0.05) within a group between a time point and day 0, while letters indicate a statistical significance (*p* < 0.05) between groups at a designated time point; (**A**) in comparison to the maternal-PBS group, (**b**) to the seronegative-oral group. ↑, immunization time points.

Before immunization,F4-specific IgG titers of pigs with maternal antibodies were significantly higher than those of F4-seronegative pigs (*p* < 0.01) (Figure 1A), In the maternal-PBS and the maternal-oral group, the IgG titers gradually declined in time, whereas in the maternal-IM group and the seronegative-oral group the IgG titer significantly increased from 0 to 21 dppi upon intramuscular or oral immunization, respectively (*p* < 0.05). Furthermore, the IgG titer in the maternal-IM group at 21 dppi was significantly higher compared to the maternal-PBS (*p* < 0.05) and the maternal-oral group (*p* < 0.01) (Figure 1A).

F4-specific IgA titers were similarly low in all 4 groups before immunization (Figure 1B). After immunization an increase in F4-specific IgA occurred in the intramuscularly immunized pigs with maternal antibodies and the orally immunized seronegative pigs. However, only in the latter this increase was statistically significant (*p* < 0.05) (Figure 1B).

### Maternal immunity enhances circulating F4-specific IgA^+^ ASC upon oral immunization

The above results suggested that maternal antibodies either suppress or mask the immune response following oral immunization. If true this should be reflected in the circulating F4-specific ASCs after immunization. When looking at the total F4-specific ASCs only significant increases were seen in the orally immunized seronegative animals and the intramuscularly immunized animals having maternal antibodies (Figure 1C). However, the primary responses seen with the ELIspot assay in both groups were clearly more obvious than when measuring the serum antibody responses. Furthermore, in the orally immunized pigs having maternal antibodies, an insignificant increase was seen in the F4-specific ASCs, similar to the increase in pigs without maternal antibodies (Figure 1C).

**Figure 2:**
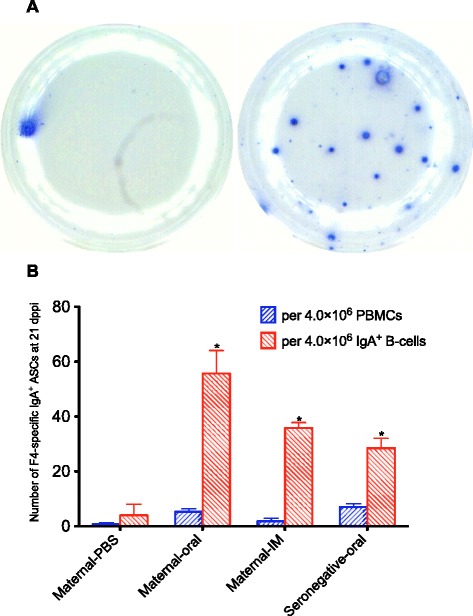
**The efficiency of using ELISpot on enriched IgA** ^**+**^ **B-cell fractions to enumerate F4-specific IgA** ^**+**^ **antibody secreting cells (ASCs).** **A** In one well, only one F4-specific IgA^+^ ASC was detected from 5 × 10^5^ peripheral blood monomorphonuclear cells (PBMCs) (left) while 18 F4-specific spots were detected from 5 × 10^5^ enriched IgA^+^ B-cells (right) at 21 days post primary immunization (dppi) of an oral immunized pig having maternal antibodies; each blue spot is counted as one F4-specific IgA^+^ ASC. **B** From eight wells, less than seven F4-specific IgA^+^ ASCs were detected from 4.0 × 10^6^ PBMCs whereas more than 28 F4-specific IgA^+^ ASCs were detected from 4.0 × 10^6^ enriched IgA^+^ B-cells at 21 dppi of the three immunized groups. The asterisk indicates a statistical significant difference (*p* < 0.05) between groups in comparison to the maternal-PBS group.

F4-specific secretory IgA induced at the gut-associated lymphoid tissue is critical to protect piglets against F4^+^ ETEC infections [14,17]. Therefore, the number of circulating F4-specific IgA^+^ ASCs in the PBMC fraction was determined. However, the number of spots in each well (0.11 ± 0.05 spots at day 0 and 0.66 ± 0.1 spots at 21 dppi per 5.0 × 10^5^ PBMCs) was too low to allow a proper analysis. This is demonstrated in Figure 2A (left); only one F4-specific spot can be seen in 5.0 × 10^5^ PBMCs at 21 dppi of an orally immunized pig with maternal antibodies. In 4 × 10^6^ PBMCs (=8 wells) of the maternal-PBS,maternal-oral,maternal-IM,seronegative-oral groups, 0.82, 5.3, 1.8 and 7 F4-specific IgA^+^ ASCs were found, respectively (Figure 2B). These numbers show no significant difference between groups. To enhance the sensitivity of the ELIspot assay for detecting IgA^+^ B cells, these cells were enriched from the PBMC fraction. In the representative picture in Figure 2A (right), 18 F4-specific spots were detected in 5.0 × 10^5^ enriched IgA^+^ B-cells. On average we could detect 4, 55.7, 35.8 and 28.5 F4-specific IgA^+^ ASCs within 4.0 × 10^6^ enriched IgA^+^ B-cells of the maternal-PBS, maternal-oral,maternal-IM,seronegative-oral pigs, respectively (Figure 2B). Thus, the enrichment increased the sensitivity of the F4-specific ELIspot assay and as such enabled the detection of differences between the groups.

As a result, the ELIspot assay on the enriched IgA^+^ cells revealed a clearly significant IgA response in the maternal-IM group at 21 dppi (*p* < 0.001) (Figure 1D). This response was similar to the F4-specific serum IgA response (Figure 1B). Also in both oral immunization groups, the ELIspot assay on the enriched IgA^+^ cells revealed a significant increase in IgA^+^ ASCs at 9 (*p* < 0.05) and 21 dppi (*p* < 0.001) (Figure 1D). Interestingly, the primary IgA^+^ ASC response upon oral immunization was similar in pigs with or without maternal antibodies (73.5 ± 43.8 spots and 63.2 ± 17.1 spots per 3.0 × 10^8^ PBMCs, respectively), whereas the secondary immune response was higher in the group with maternal antibodies than without (164 ± 28 spots versus 79 ± 16 spots per 3.0 × 10^8^ PBMCs) and even higher than in the IM immunized pigs (96 ± 1 spots per 3.0 × 10^8^ PBMCs), although these were not significantly different. Thus, in the orally immunized pigs the F4-specific IgA^+^ ASC response did not mimic the F4-specific serum IgA response (Figure 1B).

Intriguingly, the pattern of total F4-specific ASCs (Figure 1C) and F4-specific IgA^+^ ASCs (Figure 1D) within the maternal-IM group is clearly different. In this group, the number of total F4-specific ASCs is significantly higher at 9 dppi as compared to the control group, while the number of F4-specific IgA^+^ ASCs only increased at 21 dppi. This confirms that intramuscular immunization induces less IgA^+^ ASCs as compared to the other antibody subclass ASCs.

## Discussion

We demonstrated previously that oral immunization of F4-seronegative, F4 receptor positive (F4R^+^) with F4 fimbriae resulted in weak F4-specific IgG and IgA responses, which are boosted by a second immunization and lead to a protective immunity against an F4^+^ ETEC infection [3]. However, F4^+^ ETEC infections are highly prevalent and most sows are immune against this infection [2] so that most piglets obtain F4-specific antibodies via colostrum and milk. The immune response upon immunization in F4R^+^ piglets with passive immunity had not yet been evaluated. In the present study no serum antibody response was observed in pigs orally immunized in the presence of high titers of F4-specific maternal antibodies. These antibodies can obscure serum antibody responses or even suppress mucosal B cell responses, as shown in gnotobiotic pigs with high titers of passive serum antibodies against rotavirus [4,7]. To elucidate if maternal immunity masks or suppresses the serum antibody response in our study, a detection method was developed to discriminate active from passive immunity. Using ELIspot assays on PBMCs, we have previously shown that F4-specific ASC responses of orally immunized F4-seronegative pigs peaked in blood around 9 dppi [14]. In the present study, the ELIspot methodology allowed detecting immune responses in pigs orally immunized in the presence of F4-specific maternal antibodies. Interestingly, a similar response was observed in pigs with or without maternal antibodies indicating that passive immunity did not suppress the oral immunization. Since mainly sIgA is responsible for protection against an intestinal pathogen [14], the number of F4-specific IgA^+^ ASCs within the PBMC fraction was determined. Unfortunately, this number was too low to properly evaluate the immune response, due to the low frequency of antigen-specific ASC in peripheral blood as compared to mucosal tissues [18,19]. To evaluate mucosal immune responses following oral vaccination, ELIspot assays were combined with an enrichment strategy to increase its sensitivity [10,20]. Since IgA is the major isotype induced in the small intestine upon oral vaccination, we chose to enrich the circulating IgA^+^ cell fraction from blood. An alternative way might be to enrich cells expressing the intestinal homing receptor α4ß7 [10]. This integrin binds to mucosal addressin cell adhesion molecule-1 (MAdCAM-1), which is specifically expressed on endothelial cells in the intestinal lamina propria and the gut-associated lymphoid tissue [21]. However, not only B cells, but also gut-homing T cells and eosinophils express α4ß7 [22,23]. In addition, by enriching only the α4ß7^+^ cells, the immune response of the IM immunized animals, which is important in this study, would have been missed.

Comparing the ELISA and ELIspot assays confers some interesting observations. In pigs with maternal antibodies, the ELISA could only detect an F4-specific antibody response in the IM immunized animals, whereas the ELIspot assays also allowed detection of IgA^+^ B-cell response upon oral immunization. Not only the secondary response, but also the primary response was clearly observed. Furthermore, the ELIspot assay on enriched IgA^+^ B cells detected more F4-specific IgA^+^ ASCs in the presence than in the absence of maternal antibodies after the second oral immunization. Enterocytes express the human neonatal Fc receptor (FcRn), which bidirectionally transports IgG from the intestinal lumen to the underlying tissues [8,24]. Via this transport, maternal IgG could reach the gut lumen, bind antigen, and transport this back to the subepithelial antigen presenting cells (APCs) as immune complexes, leading to a priming of the immune system and protection to a challenge infection [25]. In contrast to rodents, pigs,as most other species, express FcRn throughout live [9,26,27]. We propose that FcRn transports the F4-specific maternal IgG to the gut lumen, where it binds the orally administered F4 fimbriae and transports the resulting immune complexes back to the lamina propria. There the immune complexes are endocytosed by antigen-presenting cells, such as dendritic cells, which become activated and enhance the subsequent mucosal immune response [28,29]. However, this effect is probably too weak to be observed upon the primary immunization, but might have improved priming in pigs with maternal antibodies, resulting in a stronger secondary immune response. In contrast, no enhanced priming in the presence of passive immunity, but rather suppression of the immune response, was observed in gnotobiotic pigs immunized with rotavirus-like particles or infected with rotavirus, although rotavirus-specific IgG appeared in intestinal fluid [2,5]. This discrepancy could arise from differences in antibody titers, an immature mucosal immune system in gnotobiotic pigs and the complexity of viruses and virus-like particles in comparison with soluble fimbriae.

It is know that maternal antibody titers interfere with systemic immunizations and that the ratio maternal antibodies/antigen is critical for this interference [30]. Looking at the actual levels of maternal antibodies before immunization in the maternal-oral group, titres were 160, 320, 320, 1280, 1280 and 2560. The number of F4-specific IgA^+^ ASCs in pigs with the higher maternal antibody levels reached similar amounts as those with a lower maternal antibody level ranging between 131 and 251 F4-specific IgA^+^ ASC per 3.0 × 10^8^ PBMCs, except for the piglet with the titer of 2560, which showed the lowest number F4-specific IgA^+^ ASCs after two immunizations (52 per 3.0 × 10^8^ PBMCs). Even though this low response is in accordance with interference on the immunization of high maternal antibody titers, such a conclusion cannot be drawn from an observation in only one animal

Noteworthy is the higher F4-specific serum IgA titer after intramuscular boosting as compared to the orally immunized animals, even though a smaller number of F4-specific IgA^+^ ASCs were detected in the intramuscularly immunized animals. Although the peak of the response could have been missed due to different kinetics of the ASC response following IM versus oral immunization, preliminary experiments indicate this is unlikely. Presumably, most F4-specific IgA^+^ ASCs induced by the IM immunization home to systemic tissues, whereas those induced by oral immunization home to the intestinal mucosa. In pigs, 31% of serum IgA originates from intestinal synthesis, whereas approximately 26% of intestinal IgA comes from serum [31]. Thus, measuring serum IgA only shows a fraction of the real response in orally immunized animals.

In conclusion, we clearly demonstrated that an active immune response could be induced via oral immunization of pigs with maternal antibodies. Results suggest that this maternal immunity could even enhance the systemic immune response to cognate antigen. Furthermore, results suggest that measuring numbers of circulating antigen-specific IgA^+^ ASCs within the IgA^+^ B-cells fraction from PBMCs could identify small numbers of activated antigen-specific B-cells and could be the method of choice to evaluate mucosal immune responses in pigs with maternal immunity following oral immunization.

## Abbreviations

APCs: Antigen presenting cells
ASCs: Antibody secreting cells
dppi: Days post primary immunization
F4^+^ ETEC: F4 enterotoxigenic *Escherichia coli*
F4R^+^: F4 receptor positive
FcRn: Neonatal Fc receptors
IM: Intramuscular immunization
MAdCAM-1: Mucosal addressin cell adhesion molecule-1
PBMC: Peripheral blood mononuclear cells
PBS: Phosphate buffered saline

## References

[1] Rutter JM, Jones GM (1973). Protection against enteric disease caused by Escherichia coli—a model for vaccination with a virulence determinant?. Nature.

[2] Van den Broeck W, Cox E, Goddeeris BM (1999). Seroprevalence of F4^+^ enterotoxigenic Escherichia coli in regions with different pig farm densities. Vet Microbiol.

[3] Van den Broeck W, Cox E, Goddeeris BM (1999). Receptor-dependent immune responses in pigs after oral immunization with F4 fimbriae. Infect Immun.

[4] Hodgins CD, Kang YS, De Arriba L, Parreño V, Ward AL, Yuan L, To T, Saif LJ (1999). Effects of maternal antibodies on protection and development of antibody responses to human rotavirus in gnotobiotic pigs. J Virol.

[5] Freedman DJ, Tacket CO, Delehanty A, Maneval DR, Nataro J, Crabb JH (1998). Milk immunoglobulin with specific activity against purified colonization factor antigens can protect against oral challenge with enterotoxigenic Escherichia coli. J Infect Dis.

[6] Cervenak J, Kacskovics I (2009). The neonatal Fc receptor plays a crucial role in the metabolism of IgG in livestock animals. Vet Immunol Immunopathol.

[7] Nguyen VT, Yuan L, Azevedo SPM, Jeong K, Gonzalez MA, Iosef C, Lovgren-Bengtsson K, Morein B, Lewis P, Saif LJ (2006). High titers of circulating maternal antibodies suppress effector and memory b-cell responses induced by an attenuated rotavirus priming and rotavirus-like particle-immunostimulating complex boosting vaccine regimen. Clin Vaccine Immunol.

[8] Yoshida M, Claypool SM, Wagner JS, Mizoguchi E, Mizoguchi A, Roopenian DC, Lencer WI, Blumberg RS (2004). Human neonatal Fc receptor mediates transport of IgG into luminal secretions for delivery of antigens to mucosal dendritic cells. Immunity.

[9] Stirling CMA, Charleston B, Takamatsu H, Claypool S, Lencer W, Blumberg RS, Wileman TE (2005). Characterization of the porcine neonatal Fc receptor – potential use for trans-epithelial protein delivery. Immunology.

[10] Saletti G, Cuburu N, Yang JS, Dey A, Czerkinsky C (2013). Enzyme-linked immunospot assays for direct *ex vivo* measurement of vaccine-induced human humoral immune responses in blood. Nat Protoc.

[11] Nguyen VU, Goetstouwers T, Coddens A, Van Poucke M, Peelman L, Deforce D, Melkebeek V, Cox E (2013). Differentiation of F4 receptor profiles in pigs based on their mucin 4 polymorphism, responsiveness to oral F4 immunization and in vitro binding of F4 to villi. Vet Immunol Immunopathol.

[12] Rasschaert K, Verdonck F, Goddeeris BM, Duchateau L, Cox E (2007). Screening of pigs resistant to F4 enterotoxigenic Escherichia coli (ETEC) infection. Vet Microbiol.

[13] Van den Broeck W, Cox E, Goddeeris BM (1999). Receptor-specific binding of purified F4 to isolated villi. Vet Microbiol.

[14] Van den Broeck W, Cox E, Goddeeris BM (1999). Induction of immune responses in pigs following oral administration of purified F4 fimbriae. Vaccine.

[15] Verdonck F, Snoeck V, Goddeeris BM, Cox E (2004). Binding of a monoclonal antibody positively correlates with bioactivity of the F4 fimbrial adhesin FaeG associated with post-weaning diarrhoea in piglets. J. Immunol Methods.

[16] Van Zaane D, Hulst MM (1987). Monoclonal antibodies against porcine immunoglobulin isotypes. Vet Immunol Immunopathol.

[17] McGhee RJ, Mestecky J, Dertzbaugh TM, Eldridge HJ, Hirasawa M, Kiyono H (1992). The mucosal immune system: from fundamental concepts to vaccine development. Vaccine.

[18] Czerkinsky CC, Nilsson LA, Nygren H, Ouchterlony O, Tarkowski A (1983). A solid-phase enzyme-linked immunospot (ELISPOT) assay for enumeration of specific antibody-secreting cells. J Immunol Methods.

[19] Quiding M, Nordström I, Kilander A, Anderson G, Hanson LA, Holmgren J, Czerkinsky C (1991). Intestinal immune responses in humans. Oral cholera vaccination induces strong intestinal antibody responses and interferon-gamma production and evokes local immunological memory. J Clin Invest.

[20] Lakew M, NordstrÖm I, Czerkinsky C, Quiding-Jarbink M (1997). Combined immunomagnetic cell sorting and ELISPOT assay for the phenotypic characterization of specific antibody-forming cells. J Immunol Methods.

[21] Bourges D, Zhan Y, Brady JL, Bradley H, Caminschi I, Prato S, Villadangos JA, Lew AM (2007). Targeting the gut vascular endothelium induces gut effector CD8 T cell responses via cross-presentation by dendritic cells. J Immunol.

[22] Rott LS, Briskin MJ, Andrew DP, Berg EL, Butcher EC (1996). A fundamental subdivision of circulating lymphocytes defined by adhesion to mucosal addressin cell adhesion molecule-1. Comparison with vascular cell adhesion molecule-1 and correlation with beta 7 integrins and memory differentiation. J Immunol.

[23] Wan HC, Lazarovits IA, Cruikshank WW, Kornfeld H, Center MD, Weller FP (1995). Expression of α4ß7 integrin on eosinophils and modulation of α4-integrin-mediated eosinophil adhesion via CD4. Int Arch Allergy Immunol.

[24] Dickinson BL, Badizadegan K, Wu Z, Ahouse JC, Zhu X, Simister NE, Blumberg RS, Lencer WI (1999). Bidirectional FcRn-dependent IgG transport in a polarized human intestinal epithelial cell line. J Clin Invest.

[25] Yoshida M, Kobayashi K, Kuo TT, Bry L, Glickman JN, Claypool SM, Kaser A, Nagaishi T, Higgins DE, Mizoguchi E, Wakatsuki Y, Roopenian DC, Mizoguchi A, Lencer WI, Blumber RS (2006). Neonatal Fc receptor for IgG regulates mucosal immune responses to luminal bacteria. J Clin Invest.

[26] Brambell FWR (1969). The transmission of immune globulins from the mother to the foetal and newborn young. Proc Nutr Soc.

[27] Israel EJ, Taylor S, Wu Z, Mizoguchi E, Blumberg RS, Bhan A, Simister NE (1997). Expression of the neonatal Fc receptor, FcRn, on human intestinal epithelial cells. Immunology.

[28] Regnault A, Lankar D, Lacabanne V, Rodriguez A, Thery C, Rescigno M, Saito T, Verbeek S, Bonnerot C, Ricciardi-Castagnoli P, Amigorena S (1999). Fcγ receptor–mediated induction of dendritic cell maturation and major histocompatibility complex class I–restricted antigen presentation after immune complex internalization. J Exp Med.

[29] Devriendt B, Verdonck F, Summerfield A, Goddeeris B, Cox E (2010). Targeting of Escherichia coli F4 Fimbriae to Fcγ receptors enhances the maturation of porcine dendritic cells. Vet Immunol Immunopathol.

[30] Heyman B, Wigzell H (1984). Immunoregulation by mononclonal sheep erythrocyte-specific IgG antibodies: suppression is correlated to level of antigen binding and not to isotype. J Immunol.

[31] Vaerman JP, Langendries A, Pabst R, Rothkotter HJ (1997). Contribution of serum IgA to intestinal lymph IgA, and vice versa, in minipigs. Vet Immunol Immunopathol.

